# Prevalence of Endometriosis Among Women Undergoing Laparoscopic Procedures

**DOI:** 10.1155/DTE.2.35

**Published:** 1995

**Authors:** K. K. Chu, F. P. Chen, S. D. Chang

**Affiliations:** Department of OB/GYN 222, Mai-Chin Rd. Chang Gung Memorial Hospital Keelung Branch Keelung, Taiwan China

## Abstract

We evaluated 752 patients for endometriosis in consecutive laparoscopic procedures over a one year period. Six hundred eighty patients underwent laparoscopy for indications unrelated to symptoms of endometriosis and 72 patients were diagnosed clinically to have endometriosis before the procedures. In the 72 patients with clinical indications of endometriosis, 59 patients had disease confirmed at surgery (82%). Out of 680 asymptomatic patients, 186 patients (24.7%) were documented to have the disease of various characteristics or appearances in which the typical lesions accounted for 42%. The overall prevalence among these women undergoing laparoscopy was 32.5%. However, a prevalence of 12% was shown in a subset of asymptomatic patients indicated for sterilization. This may reflect the appropriate prevalence in reproductive age population.


*Diagnostic and Therapeutic Endoscopy*, 1995, Vol. 2, pp. 35–37
Reprints available directly from the publisher
Photocopying permitted by license only

© 1995 Harwood Academic Publishers GmbH
Printed in Singapore

### Prevalence of Endometriosis Among Women Undergoing Laparoscopic Procedures

K. K. CHU, F. P. CHEN and S. D. CHANG

*Department of OB/GYN, Chang Gung Memorial Hospital, Keelung Branch, Keelung, Taiwan, R.O.C.*

*(Received June 8, 1994; in final form February 24, 1995)*

We evaluated 752 patients for endometriosis in consecutive laparoscopic procedures over a one year period. Six hundred eighty patients underwent laparoscopy for indications unrelated to symptoms of endometriosis and 72 patients were diagnosed clinically to have endometriosis before the procedures. In the 72 patients with clinical indications of endometriosis, 59 patients had disease confirmed at surgery (82%). Out of 680 asymptomatic patients, 186 patients (24.7%) were documented to have the disease of various characteristics or appearances in which the typical lesions accounted for 42%. The overall prevalence among these women undergoing laparoscopy was 32.5%. However, a prevalence of 12% was shown in a subset of asymptomatic patients indicated for sterilization. This may reflect the appropriate prevalence in reproductive age population.

**KEY WORDS:** Prevalence of endometriosis, laparoscopy, asymptomatic endometriosis, subtle endometriosis

### INTRODUCTION

Intensive research into endometriosis over the last decade has failed to elucidate its etiology and pathogenesis. There are uncertainties, too, regarding its prevalance, diagnosis and management. However, it has been estimated that endometriosis occurs in about 1–2% of women of reproductive age (1), with the incidence being 20 times higher in the infertile (2). Although the classical symptoms and signs of endometriosis are well known (3), its occurence in asymptomatic patients and absence in patients with "typical" features must not be overlooked. The prevalence of endometriosis in the general population has been underestimated by identifying the classic-morphological characteristics of endometriosis only. We now know of a range of subtle appearances that have a variable chance of being endometriosis and that may not be recognized as endometriosis by many performing laparoscopy (4). The many appearances and characteristics of endometriosis through laparoscopy were described and documented (5). Therefore, a theme of continuing interest is that of the true occurrence of the disease and it is essential that histological confirmation be obtained.

Address for correspondence: Dr. Chu Kiu-Kwong, Dept. of OB-GYN, 222, Mai-Chin Rd., Keelung, Chang Gung Memorial Hospital, Keelung, Taiwan, R.O.C.

Based on the current awareness of the numerous morphologic findings and characteristics of the condition described through laparoscopy, we attempted to further our knowledge of the prevalence of endometriosis in symptomatic and asymptomatic patients among reproductive-age women undergoing laparoscopic procedures.

### MATERIALS AND METHODS

During the one year period from January 1993 to December 1993 in Chang Gung Memorial Hospital, Keelung, the study population consisted of 752 consecutive laparoscopic procedures for various indications. All the patients indicated for the procedures were premenopausal, nonpregnant and at least 18 years old. Six hundred eighty patients underwent laparoscopic evaluation and procedures for reasons other than confirming the diagnosis of endometriosis. The remaining 72 patients were considered to have endometriosis of some kind before the procedures, having the symptoms typical of the disease, including chronic and/or cyclic pelvic pain, dysmenorrhea and deep dyspareunia. The pelvic cavity was thoroughly and systematically evaluated. The pelvic peritoneal surface was carefully inspected for subtle appearances of endometriosis (4) by employing near-contact laparoscopy. Biopsies were taken and the endometriosis severity was documented and assessed by using the revised American Fertility Society Classification (6).

### RESULTS

Out of the 752 consecutive laparoscopic procedures evaluated or performed, a total of 245 patients in which 186 were asymptomatic patients and 59 were symptomatic patients, showed evidence of endometriosis. These patients accounted for 32.5% of all women undergoing the procedures. The highest prevalence is related to the indication of pelvic adhesion in asymptomatic cases (Table 1) whereas in symptomatic patients, 50% to 91.6% were documented and compatible to the pre-evaluation diagnosis (Table 2). The majority of the asymptomatic patients which accounted for 65% had stage I disease. Only 3% of the asymptomatic cases revealed severe endometriosis. Most of them were related to menorrhagia and infertility. Common conditions which were found to have coexistent high prevalence of endometriosis were infertility, myoma and menorrhagia (Table 1). The last three conditions in Table 1 were also found to exihit high prevalence of endometriosis, but the patient number was too small. A subset of patients undergoing laparoscopic sterilization was found to have 12% prevalence of pelvic endometriosis. All patients with evidence of endometriosis presented with typical lesions in 42% and 58% were shown to exhibit subtle appearances as their lesions (Table 3).

**Table 1** Prevalence of Endometriosis Related to Indications for Laparoscopic Procedures in Asymptomatic patients

| *Indication* | *Number of patients* | *Patients with Endometriosis* | *Percentage* |
|---|---|---|---|
| Pelvic adhesion | 198 | 83 | 42% |
| Infertility | 130 | 43 | 33% |
| Myoma | 95 | 23 | 25% |
| Menorrhagia | 79 | 15 | 20% |
| Tubal pregnancy | 51 | 3 | 5% |
| Adnexal mass | 50 | 9 | 17% |
| Sterilization | 37 | 3 | 12% |
| Salpingoplasty | 14 | 2 | 17% |
| Acute pain | 13 | 1 | 7% |
| Cervical intraepithelial neoplasm | 5 | 1 | 20% |
| Urinary stress incontinence | 5 | 2 | 40% |
| Endometrial hyperplasia | 3 | 1 | 33% |

**Table 2** Prevalence of Endometriosis Related to Indications for Laparoscopic Procedures in Symptomatic patients

| *Indication* | *Number of patients* | *Patients with Endometriosis* | *Percentage* |
|---|---|---|---|
| Endometriosis | 24 | 22 | 91.6% |
| Dysmenorrhea | 15 | 13 | 86.6% |
| Adnexal mass | 8 | 6 | 75% |
| Adenomyosis | 7 | 6 | 85.7% |
| Menorrhagia | 7 | 5 | 71.4% |
| Chronic pelvic pain | 6 | 4 | 66.6% |
| Cyclic pelvic pain | 3 | 2 | 66.6% |
| Dyspareunia | 2 | 1 | 50% |

### DISCUSSION

Despite the interest endometriosis is currently attracting, it remains an elusive and confusing disease. One's view of what endometriosis is will be influenced by the context in which it is encountered. The view of the gynecologist will differ from that of the reproductive scientist or the fertility specialist. The differences of the perspectives reflect in the wide variation of the prevalence of the disease (7) and prevalence estimates vary greatly depending on the population studied (8). Most information on the distribution of endometriosis approximates prevalence data since it is based upon case series in which the number of women with endometriosis is stated as a percentage of women coming to gynecologic surgery. The prevalence ranged from 0% to 51.9% among women undergoing gynecologic operations (9,10). As awareness of the wide range of visual appearance of endometriosis is necessary to make an accurate diagnosis, many of the earlier studies may have underestimated the extent of the disease in as many as 50% of the patients (5). However, if biopsy is not employed in routine laparoscopic practice, it is possible that these appearances will be over-diagnosed as endometriosis, since they are not endometriosis in all cases (11).

In this study, pelvic adhesion is the single most frequent indication for laparoscopic procedures in asymptomatic patients. When cases are subdivided by indications for the procedures, sharp differences in prevalence estimates occur in asymptomatic (Table 1) as well as symptomatic patients (Table 2) (12–14). Considering asymptomatic

**Table 3** Type of Major Lesions and percentage Occurrence in Patients with Endometriosis

| | |
|---|---|
| Typical Black | 42% |
| White opacified peritoneum | 10% |
| Red flamelike lesions | 8% |
| Glandular lesions | 3% |
| Subovarian lesions | 21% |
| Yellow brown patches | 14% |
| Circular peritoneal defects | 2% |

patients only, the prevalence of 42% is highest in women admitted for evaluation of pelvic adhesion. Prevalence is the least for women admitted for acute pelvic pain which account for only 7%. A prevalence of 12% among patients admitted for tubal sterilization might be a group that better approximates the prevalence in the general population. However, this is far less than reported (15).

Due to the magnification of the laparoscope and video monitoring systems, it is useful in increasing the resolution of lesions which are detected. The highest occurrence of the appearances of the major lesions was the typical black lesion (Table 3) which account for 42% while the least occurrence lesion was the peritoneal defect. In subtle lesions, endometriosis was documented in 21% of the sub-ovarian lesions which all laparoscopists should pay attention to.

Epidemiologic studies may be helpful to define populations at high risk for endometriosis. As more precise estimates of the incidence or prevalence of endometriosis are obtained, it is important to establish a clear picture of the nature and history of this disease.

In conclusion, in this study population of reproductive women, the overall prevalence of endometriosis in asymptomatic patients which is 186 patients out of the group of 680 patients was 24.7%, the subset of endometriosis patients who underwent laparoscopic sterilization without symptoms of endometriosis was selected as the most appropriate reference operation on which to base a prevalence estimate of endometriosis among reproductive-age women.

### REFERENCES

1. Strathy JH, Molgaard GA, Coulam CB, et al. Endometriosis and inferility: a laparoscopic study of endometriosis among fertile and infertile women. Fertil Steril 1982;83:667–672.
2. Dmowski WP. Endometriosis as a factor in female infertility. In: Belfort P, Pinotti J, Eskes TKAB (eds.): Fertility sterility and contraception. Carmforth: Parthenon, 1989;121–124.
3. Bromham DR. Endometriosis. Update, 1989;37:14–22.
4. Janssen RP, Russel P. Non-pigmented endometriosis: clinical, laparoscopic, and pathogenic definition. Am J Obstet Gynecol 1986;155:1154–1159.
5. Martin DC, Hubert GD, Zwarg RV, et al. Laparoscopic appearances of peritoneal endometriosis. Fertil Steril 1989;51:63–70.
6. American Fertility Society: Classification of endometriosis. Fertil Steril 1985;43:351.
7. Willians TJ, Pratt JH. Endometriosis in 1000 consecutive celiotomies: Incidence and management. Am J Obstet Gynecol 1977;129:1245–1251.
8. Wheeler JM. Epidemiology of endometriosis-associated infertilty. J Reprod Med 1989;34:42–46.
9. Houston DE. Evidaence for the risk of endometriosis by age, race, and socio-economic status. Epi Reviews 1984;6:167–191.
10. Houston DE, Noller KL, Melton LJ, et al. Incidence of pelvic endometriosis in Rochester, Minnesota, 1970–1979. Am J Epi, 1987;125:959–969.
11. Nisolle M, Paindavemia B, Bowder A, et al. Histologic study of peritoneal endometriosis in infertile women. Fertil Steril 1990; 53:984–988.
12. Ripps BA, Martin DC. Focal pelvic tenderness, pelvic pain and dysmenorrhea in endometriosis. J Reprod Med 1991;36:470–472.
13. Koninckx PR, Lesaffre E, Meuleman C, et al. Suggestive evidence that pelvic endometriosis is a progressive disease whereas deeply infiltrating endometriosis is associated with pelvic pain. Fertil Steril 1991;55:759.
14. Carter JE. Combined hysteroscopic and laparoscopic findings in patients with chronic pelvic pain. J Am Assoc Gyn Lap 1994;2:43–47.
15. Moen MH. Endometriosis in women at interval sterilization. Acta Obstet Gynecol Scand 1987;66:451–454.

